# Development of a Colloidal Gold-Based Immunochromatographic Strip Targeting the Nucleoprotein for Rapid Detection of Canine Distemper Virus

**DOI:** 10.3390/bios15070432

**Published:** 2025-07-04

**Authors:** Zichen Zhang, Zhuangli Bi, Qingqing Du, Miao Zhang, Linying Cai, Yiming Fan, Jingjie Tang, Mingxing Hu, Shiqiang Zhu, Aoxing Tang, Guijun Wang, Guangqing Liu, Yingqi Zhu

**Affiliations:** 1 Shanghai Veterinary Research Institute, Chinese Academy of Agricultural Sciences (CAAS), Shanghai 200241, China; zhangzichen851005@163.com (Z.Z.); bzl601926835@163.com (Z.B.); du18639617355@163.com (Q.D.); zhangmiao_shvri@163.com (M.Z.); caicaicaiovo@163.com (L.C.); jckthrppr@alumni.sjtu.edu.cn (Y.F.); laxe914@163.com (J.T.); 19824060853@163.com (M.H.); sqzhu92@163.com (S.Z.); tangaoxing@shvri.ac.cn (A.T.); 2 College of Animal Science and Technology, Anhui Agricultural University, Hefei 230036, China; wgj2018@ahau.edu.cn

**Keywords:** canine distemper virus, colloidal gold-based immunochromatographic strip, nucleoprotein protein

## Abstract

Canine distemper, a fatal and highly transmissible disease caused by the canine distemper virus (CDV), poses a major threat to the companion animal industry. An urgent need exists for a rapid, specific, and simple method for the detection of this disease in order to improve its prevention and control. In this research, two monoclonal antibodies (mAbs), 1D3E9 and 1H9B7, were prepared, both of which specifically recognize the nucleoprotein (N protein) of CDV, and an immunochromatographic assay for CDV detection was subsequently developed using these mAbs. The results showed that both mAbs belong to the IgG1 subclass with kappa light chains. 1D3E9 was found to recognize the linear epitope ^410^AGPKQSQITFLH^421^, while 1H9B7 targeted the epitope ^450^HFNDERFPGH^459^. The test strips exhibited high specificity and good stability for up to two months when stored at 4, 25, and 37 °C. The assay exhibited a sensitivity of 10^2.39^ TCID_50_/0.1 mL. When compared with RT-PCR for detecting CDV in clinical samples, the concordance rate was 91.67%. Thus, this method shows great potential for facilitating rapid on-site detection of CDV and could be highly beneficial from the viewpoint of disease surveillance and control.

## 1. Introduction

Canine distemper (CD) is a highly infectious viral disease caused by the canine distemper virus (CDV), a member of the genus Morbillivirus within the family Paramyxoviridae. It remains a widespread and highly fatal infectious disease affecting domestic dogs, resulting in substantial economic burdens within the companion animal industry. Moreover, CDV infects a wide variety of both terrestrial and aquatic carnivores and is highly prevalent among them, posing a significant conservation threat to endangered species globally [1]. The host range of CDV spans a wide array of species, including members of the families Canidae, Procyonidae, Mustelidae, Ursidae, Viverridae, Felidae, Ailuridae, Hyaenidae, Tayassuidae, and Cercopithecidae, among others [2,3]. CD was first reported in Spain (1761), and from there, it is believed to have spread across the world [4], with one serological survey reporting high prevalence of the virus in free-ranging dog populations in many countries [5]. CDV infection induces significant immunosuppression, markedly increasing the host’s vulnerability to opportunistic infections and contributing to high morbidity and mortality rates [6]. The disease progresses with a biphasic fever and systemic involvement of the respiratory and gastrointestinal tracts, often culminating in neurological manifestations [7]. Given the rapid spread and severe disease progression caused by CDV, the development of rapid detection kits is essential.

CD was brought under control through the introduction and widespread application of live modified CDV vaccines in the 1950s [8]. However, in recent decades, there has been a re-emergence in CDV-associated infections across global canine populations, including outbreaks in vaccinated animals [9,10]. Currently, the main diagnostic techniques for CDV include virological isolation, serological assays (ELISA, indirect fluorescent antibody testing), and molecular techniques such as RT-PCR [11,12,13]. In addition, virus neutralization assays are considered the gold standard for measuring antibody titers against CDV [14]. Despite their accuracy, these diagnostic approaches are labor-intensive, time-consuming, and dependent on trained personnel and fully equipped laboratory facilities. Consequently, they are unsuitable for point-of-care or field-based applications. Given the rise in CD incidence, the need for fast, accurate, and on-site diagnostic methods for timely detection of CDV is growing.

Immunochromatographic strip (ICS) assays based on colloidal gold nanoparticles have emerged as powerful tools for point-of-care diagnostics, offering advantages such as rapidity, ease of operation, strong specificity, and direct visual interpretation without the need for specialized equipment [15]. The visible color signal generated by the aggregation of colloidal gold provides a sensitive and convenient means for detecting target analytes in various biological samples. In the context of canine distemper virus detection, ICS assays utilizing monoclonal antibodies against the viral fusion (F) envelope glycoprotein have already been developed and demonstrated promising application potential [16]. Building on this foundation, our study targets the nucleocapsid (N) protein of CDV—a highly conserved and immunogenic structural component essential for viral replication and host interaction [17]. As the earliest structural protein expressed during CDV infection, the N protein is abundantly present in virions and elicits a strong immune response in infected hosts. These characteristics make it an ideal biomarker for early and broad-spectrum detection of CDV.

Here, we report the development of a colloidal gold-based ICS assay employing N protein-specific monoclonal antibodies. This test strip demonstrates high sensitivity, strong specificity, and operational simplicity, enabling real-time, on-site detection of CDV. We believe that this rapid diagnostic tool will contribute significantly to disease surveillance and control efforts in the companion animal industry, providing a reliable platform for early intervention and outbreak prevention.

## 2. Materials and Methods

### 2.1. Viruses, Cells, and Animals

The CDV strain CDV202401 was isolated, characterized, and subsequently maintained in our laboratory. Strains of several other canine viruses, namely, adenovirus (CAV), coronavirus (CCV), parvovirus (CPV), and parainfluenza virus (CPIV), were also stored in our laboratory. Myeloma cells (SP2/0) and African green monkey kidney (Vero) cells, as experimental cell models, were cultured and maintained under our laboratory conditions. Female BALB/c mice (6–8 weeks of age) were sourced from Suzhou Xishan Biotechnology Co., Ltd. (license: SCXK (Shanghai) 2023-0004). All animal experiments conducted in this study were reviewed and approved by the Animal Care and Use Committee of the Shanghai Veterinary Research Institute, China, and were performed in accordance with humane procedures.

### 2.2. Chemicals and Reagents

All chemicals were of analytical grade and used without further purification unless otherwise specified. Chloroauric acid (HAuCl_4_) (Cat. No.: V33475) and potassium carbonate (K_2_CO_3_) (Cat. No.: 25202) were purchased from Shanghai Yuanye Bio-Technology Co., Ltd. (Shanghai, China). Bovine serum albumin (BSA) (Cat. No.: V900933) and trisodium citrate (Cat. No.: S4641) were purchased from Sigma-Aldrich (St. Louis, MO, USA). Nitrocellulose (NC) membranes, polyvinyl chloride (PVC) baseboard, bibulous paper, and glass fiber membrane were purchased from Shanghai WenXinJie Biotech Co. (Shanghai, China). Tween-20 (Cat. No.: A600560), sodium chloride (Cat. No.: A610476) and trehalose (Cat. No.: A350865) were purchased from Sangon Biotechnology (Shanghai) Co., Ltd. (Shanghai, China). All aqueous solutions were prepared using ultrapure water (Millipore, Billerica, MA, USA).

### 2.3. Plasmid Construction

The gene sequence of the CDV N protein was obtained from the CDV202401 strain. The region of the N protein with strong hydrophilicity and antigenicity was analyzed, and the region encoding amino acids of the N gene was selected. A pair of specific primers were designed for the truncated fragment: pET-28a-F: 5′-CAGCAAATGGGTCGCGGATCCatggggaacaagcctagaattg-3′, pET-28a-R: 5′-GTGGTGGTGGTGGTGCTCGAGattgagtagctctctatcat-3′. The truncated N gene was PCR-amplified, digested with Xho I and BamH I (TaKaRa Bio Inc., Shiga, Japan), and then linked to the prokaryotic expression vector pET-28a by homologous recombination (Cat No.: C116-01; Vazyme, Nanjing, China) to construct the pET-28a-N-truncated expression plasmid.

### 2.4. Recombinant Expression and Purification of Truncated N Proteins

After verifying the sequence accuracy of the recombinant plasmid pET-28a-N via enzymatic digestion and Sanger sequencing, the construct was introduced into *E. coli* BL21(DE3) for the production of the CDV N protein (Sangon, Shanghai, China). Transformed cells were cultured in LB medium containing 100 µg/mL kanamycin at 37 °C, and protein expression was induced with 1 mM IPTG (Cat. No.: I8070; Solarbio, Beijing, China) when the OD_600_ reached 0.6. After 8 h of induction, cells were harvested, lysed by sonication, and the protein was purified from the inclusion body using nickel-affinity chromatography (Cat. No.: P2247; Beyotime, Shanghai, China). Protein purity was assessed by SDS-PAGE.

### 2.5. Western Blot Identification of Recombinant Truncated N Protein

The purified recombinant protein was characterized by Western blotting. Briefly, samples were separated by SDS-PAGE (150 V, 70 min) and transferred onto a PVDF membrane (Cat. No.: 88518; Thermo Fisher, Waltham, MA, USA) using a wet-transfer system (120 V, 70 min). After blocking with 5% skim milk (Cat. No.: A600669; Sangon, Shanghai, China), the membrane was incubated overnight at 4 °C with an anti-6× His monoclonal antibody (1:1000; Cat. No.: 66005-1; Proteintech, Chicago, IL, USA), followed by an HRP-conjugated secondary antibody (1:10,000; Cat. No.: B900620; Proteintech, Rosemont, IL, USA). Signals were detected using a chemiluminescence kit (Cat. No.: SQ201; Epizyme, Shanghai, China).

### 2.6. Animal Immunization Strategy and Selection of Hybridoma Cells Producing mAbs Against the Truncated N Protein

Five eight-week-old female BALB/c mice were subcutaneously immunized with purified truncated N protein emulsified in complete Freund’s adjuvant (Cat. No. F5881; Sigma, St. Louis, MO, USA), followed by two booster injections with incomplete Freund’s adjuvant (Cat. No. F5506; Sigma, USA) at two-week intervals. One week following the final booster, tail vein blood was collected to assess serum antibody titers via indirect ELISA. The mouse exhibiting the highest antibody response was selected for an intraperitoneal booster immunization.

Spleen cells were isolated from immunized mice three days after the final booster and fused with SP2/0 myeloma cells to generate hybridomas. Clones secreting antibodies specific to the recombinant N protein were identified through multiple rounds of ELISA screening and subcloning. Stable hybridoma lines producing high-affinity mAbs were subsequently expanded and cryopreserved for long-term storage.

### 2.7. Preparation of Mouse Ascites and Purification of mAbs

Immunized BALB/c mice aged 8 to 10 weeks were administered 500 µL of paraffin oil (Cat. No.: B500301; Sangon, Shanghai, China) intraperitoneally to induce ascites formation, thereby efficiently collecting monoclonal antibodies at high concentrations. One week later, a 500 µL suspension of hybridoma cells at a concentration of 1 × 106 to 6 × 106 cells/mL was injected into each mouse. When the mice exhibited signs of abdominal enlargement, rough fur, and slow movement, ascitic fluid was collected. Following the instructions of the r Protein G chromatography medium manual, the obtained ascitic fluid was purified, and the target antibodies were collected and stored at −80 °C.

### 2.8. Western Blot Identification of mAbs

Vero cells were cultured in a 6-well plate until reaching approximately 70% confluency, and then CDV virus solution was inoculated. After 2 h incubation at 37 °C under 5% CO_2_, the maintenance culture medium (2% FBS) was replaced and incubated for an additional 24 h before cells were collected to prepare the samples. Samples were analyzed by Western blotting according to the previously described method.

### 2.9. Validation of mAbs Using Indirect Immunofluorescence Assay

Vero cells were seeded in 12-well plates and infected with CDV. When cytopathic effects reached ~50%, cells were fixed with 4% paraformaldehyde and permeabilized with cold methanol. After blocking with 5% skim milk, purified ascitic fluid containing the two mAbs was applied as the primary antibody, with a blank control included. Cells were then incubated with FITC-conjugated goat anti-mouse IgG (1:1000; Cat. No.: A-11001; Thermo Fisher, USA), followed by DAPI nuclear staining (Cat. No.: C1005; Beyotime, Shanghai, China). Fluorescence images were acquired using a fluorescence microscope.

### 2.10. Preliminary Identification of the Antigenic Epitope of the Generated mAb

The N protein was sequentially truncated into four overlapping fragments, and specific primers were designed for each fragment (primers are shown in Table 1). PCR-amplified fragments were subcloned into the pEGFP-C3 vector to construct recombinant plasmids pEGFP-C3-N1, pEGFP-C3-N2, pEGFP-C3-N3, and pEGFP-C3-N4, which were transfected into well-grown 293T cells. Protein samples were harvested 24 h post-transfection and analyzed by Western blotting to map the antigenic regions of the truncated CDV N protein recognized by the mAbs. To further identify the precise epitopes, the corresponding peptides were progressively truncated until the exact binding sites of the monoclonal antibodies were identified.

### 2.11. Synthesis of Colloidal Gold and Optimization of Labeling Conditions

Colloidal gold nanoparticles were synthesized using the classical citrate reduction method. Briefly, 100 mL of 0.01% (*w*/*v*) chloroauric acid solution (HAuCl_4_·3H_2_O) was heated to boiling under vigorous stirring. Then, 1.5 mL of 1% (*w*/*v*) trisodium citrate solution was rapidly added. The solution was kept boiling for 10 min. During the reaction, the color gradually changed from pale yellow to colorless, gray, and finally wine red, indicating the formation of colloidal gold. The solution was cooled to room temperature and stored at 4 °C for further use. The morphology and size distribution of the synthesized colloidal gold nanoparticles were examined using transmission electron microscopy.

pH is a key factor in antibody–gold conjugation, as it affects both nanoparticle stability and antibody integrity [18,19,20]. An optimal pH promotes efficient antibody adsorption onto negatively charged gold surfaces while avoiding aggregation or antibody denaturation. To optimize the pH for antibody conjugation, 1 mL of colloidal gold solution was adjusted to various pH values by adding 0.2 mol/L K_2_CO_3_. A fixed amount of antibody was then added and incubated at room temperature for 15 min, followed by the addition of NaCl (final concentration: 1%). The color stability of the solution was observed. The optimal pH was determined based on a combination of UV–Vis absorption spectra, visual color changes, and transmission electron microscopy (TEM) observations of particle morphology. A pH range of 8.5–9.0 resulted in a stable surface plasmon resonance peak (~520 nm), consistent red coloration, and well-dispersed spherical nanoparticles, indicating good colloidal stability for antibody conjugation.

To determine the optimal antibody amount, different amounts of monoclonal antibody (0–20 μg) were added to 1 mL of colloidal gold solution at the optimal pH. After incubation for 15 min, 100 μL of 10% NaCl was added to each tube. The minimum antibody amount that prevented aggregation and maintained the red color of the solution was considered optimal. In this study, 14 μg/mL was selected as the optimal antibody concentration for labeling.

### 2.12. Preparation of Colloidal Gold–Labeled Antibody

Gold-labeled antibodies were prepared under the optimized pH and antibody concentration conditions. The monoclonal antibody (14 μg/mL) was added to colloidal gold solution adjusted to pH 8.91, and the mixture was gently stirred and incubated at room temperature for 30 min to allow full adsorption. Subsequently, bovine serum albumin (BSA) was added to a final concentration of 1% to block nonspecific binding sites on the gold surface, followed by a further 15 min of incubation.

The mixture was centrifuged at 12,000× *g* for 30 min at 4 °C to remove excess antibody and unbound proteins. The pellet containing antibody-conjugated gold nanoparticles was then resuspended in PBS (pH 7.4) and washed twice by repeated centrifugation under the same conditions to further eliminate residual unbound antibodies. The final pellet was resuspended in storage buffer and stored at 4 °C until use.

The size of the gold nanoparticles (AuNPs) and antibody-conjugated AuNPs (mAb–AuNPs) was determined from TEM images using ImageJ software (version 1.53, National Institutes of Health, Bethesda, MD, USA). The scale bar in each TEM image was used for pixel-to-nanometer calibration. A total of 100 particles per group were manually analyzed using the Feret diameter measurement function. The data were used to generate histograms of the size distribution, and results were expressed as mean ± standard deviation.

### 2.13. Preparation and Assembly of the Colloidal Gold ICS

The lateral flow strip was constructed with an absorbent pad, nitrocellulose membrane, gold conjugate pad, and sample pad mounted on a plastic backing. Monoclonal antibodies and goat anti-mouse IgG were pre-coated at the test (T) and control (C) lines, respectively. Finished strips were cut to 4 mm width, assembled into cassettes, and stored in dry packaging.

For analysis, samples were combined with colloidal gold-conjugated monoclonal antibodies and applied to the sample pad. In positive specimens, CDV antigen-gold antibody complexes migrated via capillary action to the T line, forming a visible red band through nanoparticle aggregation. Excess gold conjugates bound to goat anti-mouse IgG at the C line, validating test integrity. The simultaneous appearance of both T and C line bands confirmed a positive result, while the C line alone indicated validity with a negative outcome.

### 2.14. Specificity and Sensitivity Assessment of the Colloidal Gold Immunochromatographic Strip (ICS)

CDV at a titer of 104.5 TCID50/mL was diluted in a series (1:2, 1:4, 1:8, 1:16, 1:32, 1:64, 1:128, and 1:256) and then tested. The strips generated were then used to test the CDV-infected Vero cell culture and untreated Vero cell culture, and the results were recorded after allowing the strips to sit at room temperature for 5 min. To assess specificity, the strips were also tested on CAV-, CPIV-, CPV-, and CCV-infected cells.

### 2.15. Repeatability and Stability of the Colloidal Gold ICS

Assessment of assay reproducibility was performed by analyzing CDV detection across different batches of test strips. In order to determine the effects of storage under various conditions, the prepared test strips were placed at 4 °C, room temperature, and 37 °C for 7 d, 30 d, and 60 d, and then tested again for their ability to detect CDV. To evaluate storage stability under diverse environmental conditions, test strips were stored at 4 °C, ambient temperature (25 °C), and 37 °C for 7-, 30-, and 60-day intervals, with subsequent CDV detection capability through standardized operation protocols.

### 2.16. Clinical Application of the Colloidal Gold ICS

Peripheral blood and nasal swab samples were collected from 24 dogs clinically suspected of CDV infection. The samples were tested using the developed colloidal gold test strips, and the results were compared with those obtained through RT-PCR to assess the accuracy of the test strips.

### 2.17. Biological Information Analysis

Epitope characterization was conducted using Jalview (version 2.11, University of Dundee, Dundee, Scotland, UK) and PyMOL (version 2.5, Schrödinger, LLC, New York, NY, USA). The mapping of the identified epitope on the CDV N protein was visualized with PyMOL molecular visualization (v2.5.2) using a homology-modeled tertiary structure. Sequence alignment was performed in MEGA 7 using the Clustal W algorithm, and the phylogenetic tree based on the CDV N gene was constructed using the Neighbor-Joining method with the software MEGA (version 11, Temple University, Philadelphia, PA, USA). To assess epitope conservation, the identified region was aligned with N protein sequences from diverse CDV strains using Jalview. All reference sequences and associated epidemic strain data were obtained from the NCBI database.

## 3. Results

### 3.1. Construction of the Truncated N Protein Expression Plasmid and Purification of the Recombinant Protein

**Figure 1:**
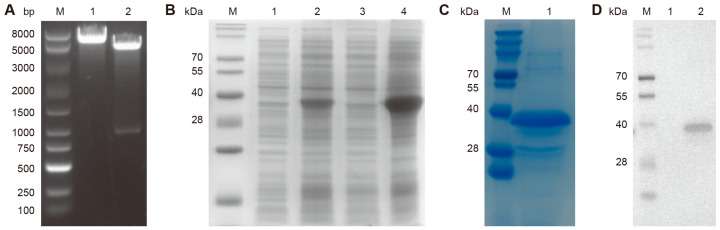
Identification of recombinant plasmids and recombinant proteins. (**A**) Restriction enzyme identification of the recombinant plasmid pET28a-truncated-N. Lane 1 represents the bands that developed following single enzyme digestion of the recombinant plasmid, and lane 2 represents the results of double enzyme digestion of the recombinant plasmid. (**B**) SDS-PAGE analysis of the recombinant plasmid expression induced by IPTG. Lanes 1, 2, 3, and 4 represent the bands obtained after solubility analysis of the total bacterial component without IPTG induction, total bacteria after IPTG induction, supernatant of the bacterial lysate after IPTG induction, and precipitates of the bacterial lysate after IPTG induction, respectively. (**C**) SDS-PAGE results showing the purified N protein. (**D**) Western blot analysis of the recombinant protein (lane 2) using an anti-His-tagged antibody, with lane 1 representing the empty vector pet-28a as a negative control.

A recombinant plasmid, designated pET28a-truncated-N, was constructed by inserting the truncated N gene into the multiple cloning site of the pET28a vector. Double digestion using BamHI and XhoI resulted in the development of two distinct bands on the electrophoresis gel, which were considered to represent the 5334 bp expression vector and the 840 bp target band, respectively (Figure 1A). As shown in Figure 1B, the truncated-N protein was successfully expressed after IPTG induction. Solubility analysis revealed predominant localization of the recombinant protein in inclusion bodies (Figure 1B), and SDS-PAGE confirmed high purity after purification (Figure 1C). Western blot further demonstrated specific binding of the recombinant protein to the His-tagged monoclonal antibody (Figure 1D). These findings confirm the effective expression, purification, and antigenic functionality of the recombinant N protein and can serve as an antigen for mouse immunization to generate monoclonal antibodies.

### 3.2. Development and Validation of Monoclonal Antibodies Against the CDV Recombinant N Protein

**Figure 2:**
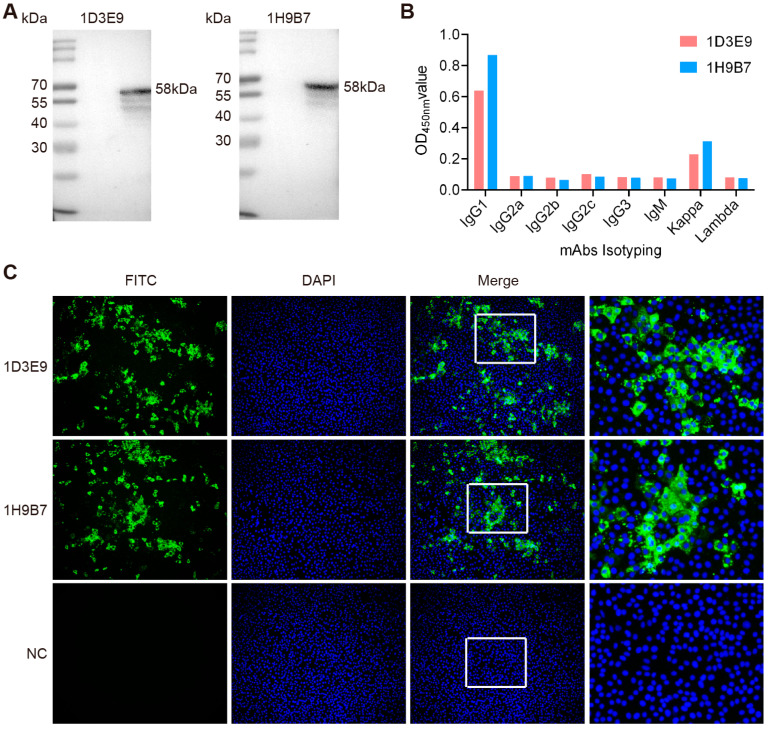
Characterization of the isolated mAbs against CDV. (**A**) Western blots showing the specificity of the two mAbs against the N protein in CDV-infected Vero cells. (**B**) Subtype analysis of mAbs. (**C**) Indirect immunofluorescence assay depicting the fluorescence expressed by the two monoclonal antibodies in Vero cells infected with CDV. As shown in the negative control panels, no immunofluorescence was observed in Vero cells that were not infected with CDV. Enlarged views of the boxed areas are shown in the right panels.

Following immunization with the truncated N protein, mice developed a robust humoral response, with serum antibody titers reaching 1:64,000 after three doses, as determined from tail blood samples. Following immunization, spleen cells from mice were fused with SP2/0 myeloma cells to establish hybridomas. Screening of the resulting hybridoma cells was performed using indirect ELISA combined with limiting dilution cloning. Through this process, two stable hybridoma cell lines, designated 1D3E9 and 1H9B7, were successfully established and shown to secrete monoclonal antibodies specifically targeting the CDV N protein. Western blot results showed strong reactivity between the mAbs and CDV, confirming the specificity of their ability to recognize the N protein (Figure 2A). Isotype characterization demonstrated that both monoclonal antibodies belonged to the IgG1 subclass and possessed kappa light chains (Figure 2B). Furthermore, immunofluorescence staining revealed clear, localized fluorescence in CDV-infected Vero cells, confirming the specific binding of these antibodies to the viral N protein (Figure 2C).

### 3.3. Epitope Recognition Ability of the mAbs and Spatial Location of the Epitope

**Figure 3:**
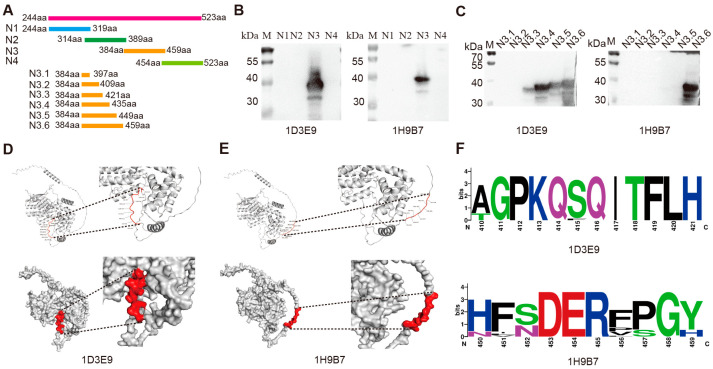
Identification and characterization of the N protein epitopes recognized by the mAbs. (**A**) Schematic representation of truncated CDV N protein fragments used for the identification of the mAbs epitopes. (**B**) Preliminary identification of the antigenic epitopes recognized by two mAbs. (**C**) Fine mapping of the antigenic epitopes recognized by two monoclonal antibodies. (**D**,**E**) Visualization of the antigenic epitopes 1D3E9 and 1H9B7. The upper panels illustrate the backbone trace of the antigen peptide, with red segments highlighting the key epitope residues recognized by each antibody. The lower panels present the surface representations of the antigen, where the antibody-binding epitopes are shown in red on the gray molecular surface. Dashed boxes indicate the zoomed-in regions to visualize the spatial orientation of the antibody-epitope interface in greater detail. (**F**) Graphical representation of the two epitopes recognized by the mAbs 1D3E9 (410–421aa) and 1H9B7 (450–459aa) exhibiting a high degree of conservation.

The N protein was separated into the overlapping fragments N1, N2, N3, and N4 (shown in Figure 3A), and Western blot analysis of these fragments indicated that the antigenic epitopes were all located in the N3 segment (Figure 3B). Therefore, further truncation of the N3 segment was carried out to construct the recombinant plasmids pEGFP-C3-N3.1, pEGFP-C3-N3.2, pEGFP-C3-N3.3, pEGFP-C3-N3.4, pEGFP-C3-3.5, and pEGFP-C3-N3.6, which were used to overexpress the proteins in 293T cells. Western blot results showed that the mAb 1D3E9 recognized an antigenic epitope located at segment N3.3, while the mAb 1H9B7 recognized an antigenic epitope located at segment N3.6 (Figure 3C). Analysis of the spatial structure of the epitopes with PyMol showed that the linear epitopes ^410^AGPKQSQITFLH^421^ and ^450^HFNDERFPGH^459^ were surface-exposed on the N protein (Figure 3D,E). Alignment was performed on the N protein amino acid sequences obtained from CDV strains of both domestic and international origin. A WebLogo was generated using the WebLogo 3 website, and the graphical representation indicated that the epitopes of the two mAbs exhibited a high degree of conservation (Figure 3F).

### 3.4. Characterization of Epitope Conservation Among CDV Strains and Their Recognition by Monoclonal Antibodies

**Figure 4:**
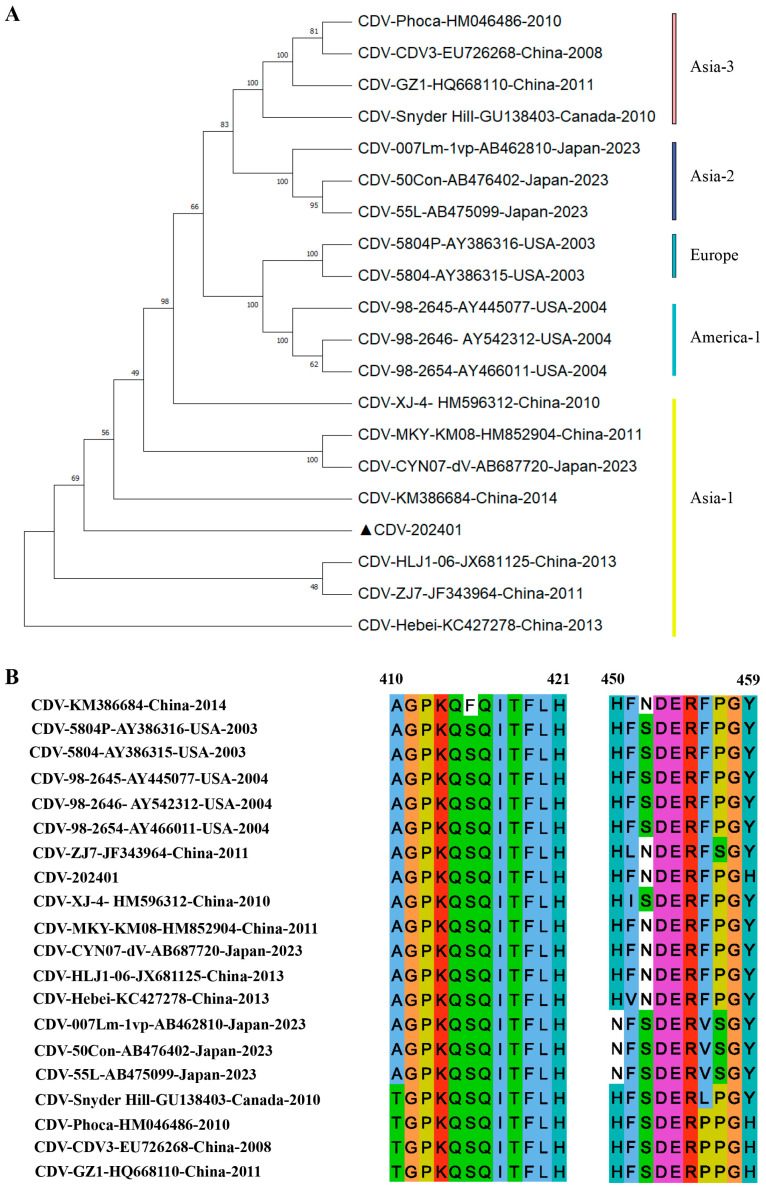
Conservation analysis of the identified epitopes among different CDV epidemic isolates. (**A**) Phylogenetic tree of the 20 representative CDV strains cited in this research. The strains marked with ▲ were the ones used in this study. (**B**) Degree of conservation of the epitopes ^410^AGPKQSQITFLH^421^ and ^450^HFNDERFPGH^459^ across the 20 CDV strains.

A selection of 20 classical CDV strains, representing the most relevant isolates, was subjected to sequence analysis. The amino acid sequences of their N proteins were aligned using MEGA-X software. Phylogenetic analysis revealed that the strain CDV202401 employed in this study clustered within the Asia-1 genotype (Figure 4A). Notably, the identified epitope, spanning residues 410–421 (AGPKQSQITFLH), exhibited a high degree of conservation across all analyzed CDV lineages, suggesting its potential role as a broadly conserved B-cell epitope suitable for universal diagnostic or therapeutic applications (Figure 4B). However, only four amino acids of the ^450^HFNDERFPGH^459^ epitope were highly conserved (Figure 4B). This suggests that the epitope of 1H9B7 may serve as an important marker associated with viral virulence or may be involved in the genotypic classification of CDV.

### 3.5. Colloidal Gold–Antibody Conjugate Preparation

**Figure 5:**
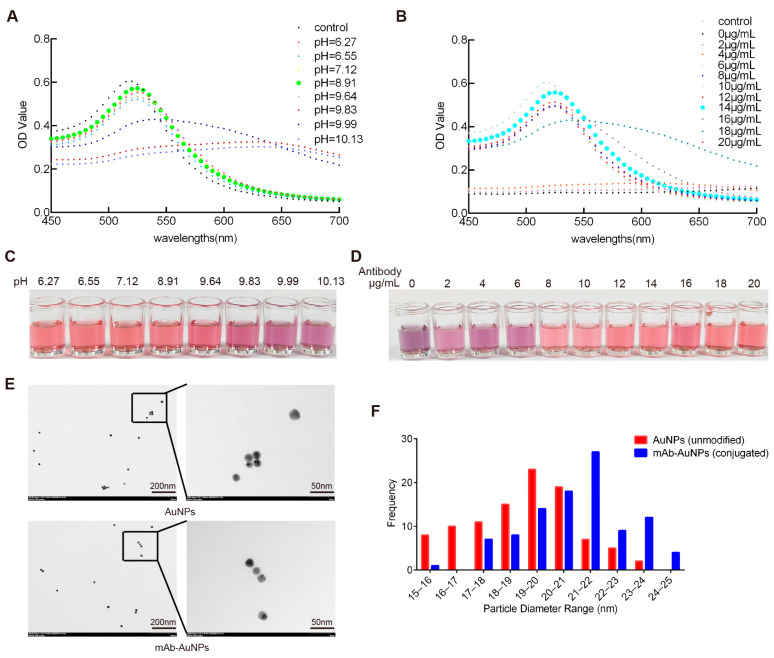
Preparation of colloidal gold probes. (**A**) Absorption curves corresponding to the tested volumes of 0.2 mol/L K_2_CO_3_ solution. (**B**) Absorption curves corresponding to the tested antibody dosages. (**C**) Visual color changes of AuNP solutions under different pH conditions (6.27–10.13). (**D**) Colloidal gold solutions obtained with the addition of antibody dosages of 0, 2, 4, 6, 8, 10, 12, 14, 16, 18, and 20 μg/mL. (**E**) Transmission electron microscopy (TEM) analysis of AuNPs and mAb-AuNPs. (**F**) Histogram of unmodified AuNPs and mAb–AuNP conjugates. Particle size was determined from TEM images using ImageJ software version 1.53. A total of 100 particles were measured per group based on Feret diameter.

To optimize the pH and antibody concentration for conjugation, we systematically evaluated the colloidal stability and spectral behavior of the gold nanoparticles under various conditions. UV–Vis spectra revealed a characteristic surface plasmon resonance peak at ~520 nm (Figure 5A), which was most stable at pH 8.5–9.0. At lower pH values, the absorbance decreased and broadened, reflecting partial aggregation. Visual inspection also supported these findings, with optimal red color observed in the pH 8.5–9.0 range (Figure 5C). In antibody titration experiments, increasing antibody concentration led to enhanced stabilization of the AuNPs (Figure 5B). A plateau in absorbance was observed above 14 μg/mL, suggesting surface saturation and minimal aggregation. Corresponding visual color changes were consistent, with deep red maintained at higher antibody concentrations (Figure 5D). As shown in Figure 5E, TEM imaging confirmed that the synthesized AuNPs were spherical and well dispersed. After antibody conjugation, no obvious aggregation was observed, indicating good colloidal stability. The average particle size of unmodified AuNPs was 19.08 ± 1.90 nm, while that of antibody-conjugated AuNPs was 20.89 ± 1.94 nm, indicating a moderate increase in diameter after conjugation. This change in size is consistent with the formation of a protein corona and confirms successful surface functionalization. As shown in Figure 5F, histograms of the particle size distribution further demonstrate the overall shift in size following bioconjugation.

### 3.6. Evaluation of the Specificity and Sensitivity of the Immunochromatographic Strips

**Figure 6:**
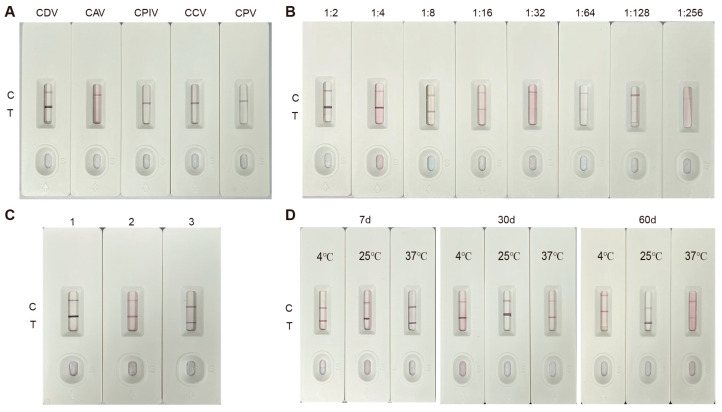
Evaluate the specificity, sensitivity, and stability of the colloidal gold immunochromatographic strip. (**A**) Specificity testing of the test strips against various canine viruses indicating specificity for CDV. (**B**) Sensitivity testing of the strips with various dilutions of CDV. (**C**) Repeatability of the test determined using strips generated in different batches. (**D**) Stability testing of the strips under various temperature conditions and storage times.

The colloidal gold strips test showed a positive result for Vero cells infected with CDV and a negative result for uninfected Vero cells. The test strip results were also negative for cells infected with CAV, CPIV, CCV, and CPV (Figure 6A). These findings demonstrate the high specificity of the strips. The colloidal gold test strip was used to detect CDV at various dilutions, and the lowest detectable amount of the CDV antigen was 10^2.39^ TCID_50_/0.1 mL (Figure 6B). The visual limit of detection (LOD) for CDV was determined to be 10^2.39^ TCID_50_/0.1 mL, as this was the lowest concentration at which ≥95% of replicates (19/20) produced a clearly visible test line. At lower concentrations, the positive rate dropped below the 95% threshold. The average visual score at this concentration also exceeded the predefined threshold of 1.0, supporting its designation as the visual LOD (Supplementary Figure S1 and Table S1). This finding confirms the strip’s high sensitivity for CDV identification and its excellent specificity, as no cross-reactivity with other canine viruses was observed.

### 3.7. Repeatability and Stability of the CDV Test Strips

In order to assess the repeatability of the colloidal gold test strips, different batches were employed to detect CDV-infected Vero cell supernatants. Consistent positive results were obtained across batches, demonstrating the strips’ reliable repeatability (Figure 6C). The packaged colloidal gold test strips were kept at 4 °C, room temperature, and 37 °C, for different time periods (7, 30, and 60 days) and then used to detect CDV in the supernatant of infected Vero cells. The results were all positive, indicating that the test strips have good stability (Figure 6D).

### 3.8. Evaluation of the Consistency Between Colloidal Gold Test Strips and RT-PCR in Detecting CDV

**Figure 7:**
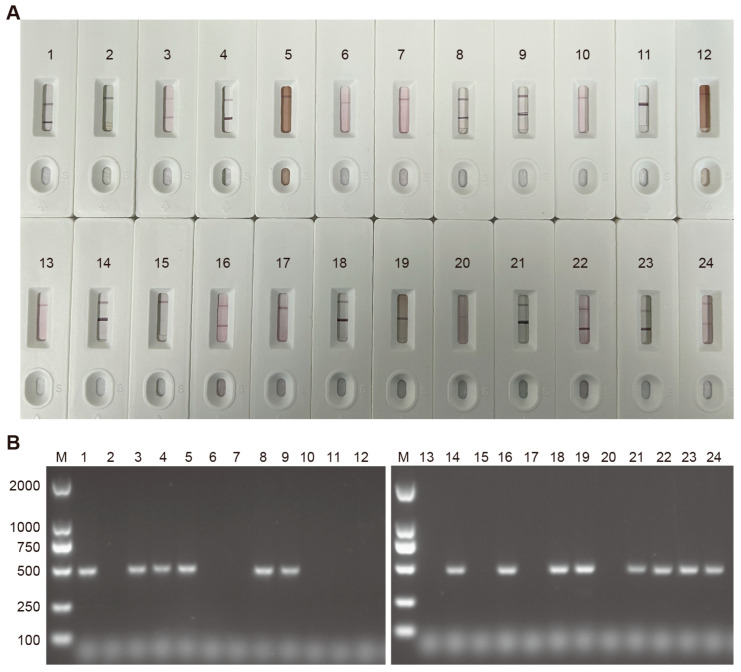
Detection of CDV in suspected samples with the colloidal gold strip test and RT-PCR. (**A**) Colloidal gold test strips for examining suspected samples. (**B**) RT-PCR testing.

The results of the colloidal gold test strips showed that out of 24 samples of suspected CDV infection, 12 were infected with CDV (Figure 7A). RT-PCR analysis of 24 clinical samples revealed 14 positive cases (Figure 7B). Similar results were obtained with both tests for 22 out of the 24 samples, indicating a concordance rate of 91.67%.

## 4. Discussion

In this study, a rapid immunochromatographic strip for CDV detection was successfully established. Comprehensive evaluations demonstrated that the strip assay possesses high specificity, sensitivity, repeatability, and stability, thereby offering a reliable and convenient tool for on-site, real-time diagnosis of CDV infections.

Colloidal gold-labeled immunochromatographic strip (ICS) assays have demonstrated significant utility in the clinical detection of viruses, bacteria, pesticides, and pharmaceutical residues [21,22,23]. Their widespread application can be attributed to several inherent advantages, including rapid diagnosis, high sensitivity, cost-effectiveness, and operational simplicity [24]. Research has shown that colloidal gold immunochromatographic assays enable faster detection of viral IgM or IgG antibodies compared to real-time RT-PCR assays. Therefore, colloidal gold-labeled ICS assays are highly recommended as a supplementary diagnostic approach in the clinical setting [25]. A key advantage of this assay lies in its rapid turnaround time, as the entire detection process can be completed within 15 min without the need for specialized instrumentation. Moreover, in the early phase of canine infection, conjunctival swabs can be non-invasively obtained, which further enhances the assay’s practicality for point-of-care and field-based diagnostics. Similar to these previous findings, our test results showed 91.67% concordance between the results obtained with the test strips and RT-PCR. Moreover, our colloidal gold-based detection can be completed within 15 min, whereas the RT-PCR method requires a full day. The prepared colloidal gold test strips could specifically detect only CDV and did not react with CAV, CPV, CPIV, CCoV, and other pathogens. Strips from different batches showed similar results, indicating the repeatability of the test, and their CDV detection ability was not affected by storage for up to 60 days and temperatures of 4 °C and 37 °C. Overall, these results indicate that the prepared ICS assay combines high sensitivity, strong specificity, good stability, and reliable repeatability with a simple and rapid detection procedure, highlighting their promising potential for clinical application.

A significant contribution of this study is the development of two monoclonal antibodies specifically targeting the CDV N protein, which, in contrast to the more commonly used H or F proteins, has rarely been employed as a diagnostic target in immunochromatographic test strips. This highlights the novelty of our approach and expands the potential repertoire of diagnostic targets for CDV detection. While multiple proteins contribute to CDV pathogenesis, the N protein stands out due to its exceptional abundance and superior capacity to trigger antibody production [26]. Moreover, the N protein is exposed in the process of virus assembly, and this makes it a suitable target of clinical detection [27]. Indeed, the N protein of virions has been used to establish assays in several studies on other pathogenic viruses though. For instance, the feasibility of detecting the N protein of Middle East Respiratory Syndrome Coronavirus (MERS-CoV) using antigen-based diagnostic approaches has been demonstrated [28]. Similarly, a newly developed panel of monoclonal antibodies targeting the nucleoprotein and glycoprotein Gc of Schmallenberg virus enabled specific detection of orthobunyaviruses [29]. Furthermore, ELISA detection methods based on viral nucleoprotein have been reported in a wider range of other viruses, including SARS-CoV-2 [30], Crimean-Congo Hemorrhagic Fever Virus [31], Peste des Petits Ruminants Virus [32], and influenza virus [33], among others. This study highlights the potential of the CDV N protein as a viable target for immunodiagnostic applications and provides a foundation for developing colloidal gold-based test strips targeting the nucleocapsid proteins of other viral pathogens.

Although monoclonal antibodies against the CDV N protein have been developed [17,34,35,36], the precise linear epitopes remain largely uncharacterized. Therefore, detailed mapping of these epitopes and analysis of their antigenic variability are essential for identifying conserved and variable regions of the N protein across different viral strains. In this research, we mapped the precise linear epitopes of the two mAbs of N protein using the Western blot analysis with overlapping peptides that covered the whole region of truncated N protein. Two novel linear epitopes, ^410^AGPKQSQITFLH^421^ and ^450^HFNDERFPGH^459^, were identified and recognized by the monoclonal antibodies 1D3E9 and 1H9B7, respectively. The peptide epitope ^410^AGPKQSQITFLH^421^ targeted by mAb-1D3E9 is highly conserved among various CDV lineages, suggesting its potential as a universal B-cell epitope in CDV. Structural prediction and analysis of this linear epitope (^410^AGPKQSQITFLH^421^) using Jalview and PyMOL revealed its surface-exposed localization within the N protein. This finding aligns well with indirect immunofluorescence assay (IFA) results, which show that mAb-1D3E9 specifically recognizes the N protein in CDV-infected cells, suggesting this epitope as a potential universal target for CDV detection across all lineages. However, the linear epitope targeted by the 1H9B7 mAb is characterized by a low degree of sequence conservation across circulating CDV strains. This suggests that the epitope may serve as an important marker associated with viral virulence or may be involved in the genotypic classification of CDV. The specific function of the 1H9B7 linear epitope remains to be further explored. The identification of these novel epitopes will enhance our understanding of the immunological characteristics and roles of the CDV N protein while facilitating the development of N protein epitope-based strategies for CDV infection prevention and diagnosis.

## 5. Conclusions

In conclusion, two monoclonal antibodies specific to CDV were successfully generated and employed in the development of a colloidal gold lateral flow assay, enabling rapid CDV detection. This assay, which yields results within 15 min and requires neither specialized equipment nor trained personnel, holds significant promise for enhancing CDV surveillance. It may also facilitate timely diagnosis and contribute to the implementation of more effective prevention and control strategies for CDV, both in China and globally.

## Figures and Tables

**Table 1:** Sequences of primers used for truncated CDV N proteins.

Primers	Sequence (5′-3′)
pEGFP-C3-N1-F	TACAAGTACTCAGATCTCGAGTTGGCATCACCAAG
pEGFP-C3-N1-R	CGACTGCAGAATTCGAAGCTTAGAATTTTCCAGAATAACCAT
pEGFP-C3-N2-F	TACAAGTACTCAGATCTCGAGGTTATTCTGGAAAATTCTGTTCAG
pEGFP-C3-N2-R	CGTCGACTGCAGAATTCGAAGCTTTTCCTTGGTGATGCC
pEGFP-C3-N3-F	TACAAGTACTCAGATCTCGAGCTTGGCATCACCAAG
pEGFP-C3-N3-R	CCGTCGACTGCAGAATTCGAAGCTTGTGCCCTGGAAAGCG
pEGFP-C3-N4-F	TACAAGTACTCAGATCTCGAGGAACGGTTTTCCAGG
pEGFP-C3-N4-R	CGTCGACTGCAGAATTCGAAGCTTGATGGTTGGGGGTTGTTG
pEGFP-C3-N3.1-F	TACAAGTACTCAGATCTCGAGCTTGGCATCACCAAG
pEGFP-C3-N3.1-R	CGTCGACTGCAGAATTCGAAGCTTTATTTCTGGCACTAGC
pEGFP-C3-N3.2-R	CCGTCGACTGCAGAATTCGAAGCTTAGTGCGAATCGTCCGGT
pEGFP-C3-N3.3-R	CCGTCGACTGCAGAATTCGAAGCTTGTGCAGAAAAGTGAT
pEGFP-C3-N3.4-R	CGTCGACTGCAGAATTCGAAGCTTGATGGTTGGGGGTTGTTG
pEGFP-C3-N3.5-R	CCGTCGACTGCAGAATTCGAAGCTTGATGGGGTATTTGTC
pEGFP-C3-N3.6-R	CGTCGACTGCAGAATTCGAAGCTTGATGGTTGGGGGTTGTTG

## Data Availability

The raw data supporting the conclusions of this article will be made available by the authors on request.

## Supplementary Materials

The following supporting information can be downloaded at: https://www.mdpi.com/article/10.3390/bios15070432/s1, Table S1: Visual detection rates at serial CDV concentrations. Each concentration of CDV was tested in 5 replicates, except for 10^2.39^ TCID_50_/0.1 mL, which was tested in 20 replicates. A visual positive was defined as the presence of a clearly visible test line as judged independently by three trained observers. The visual positive rate was calculated as the percentage of positive readings among total replicates. Mean score refers to the average visual intensity score assigned by observers on a scale from 0 (no line) to 2 (strong line). The visual limit of detection (LOD) was defined as the lowest concentration at which ≥95% of replicates showed visible bands, with an average score ≥ 1.0 across replicates (10^2.39^ TCID_50_/0.1 mL in this case). Figure S1: Determination of the visual limit of detection (LOD) for CDV using serial dilutions. Visual detection rates and average visual scores for CDV at serial 2-fold dilutions. A visual positive was defined as a clearly visible test line evaluated independently by three observers. Each dilution was tested in five replicates, except for the critical LOD concentration (10^2.39^ TCID_50_/0.1 mL), which was tested in 20 replicates to improve statistical confidence. The blue dashed line represents the 95% visual positivity threshold. The red dashed line indicates the visual score threshold of 1.0, above which the test line was generally distinguishable by eye. The visual LOD was defined as the lowest concentration at which ≥95% of replicates yielded a visually positive result.

## Abbreviations

The following abbreviations are used in this manuscript:

Abbreviations	Meaning
CDV	Canine Distemper Virus
CD	Canine Distemper
mAb	Monoclonal Antibody
N protein	Nucleoprotein
ELISA	Indirect Fluorescent Antibody Testing
ICS	Immunochromatographic Strip
CAV	Canine Adenovirus
CCV	Canine Coronavirus
CPV	Canine Parvovirus
CPIV	Canine Parainfluenza Virus
MERS-CoV	Middle East Respiratory Syndrome Coronavirus
IFA	Indirect Immunofluorescence Assay
