# Supplementary material for: Development of a Colloidal Gold-Based Immunochromatographic Strip Targeting the Nucleoprotein for Rapid Detection of Canine Distemper Virus
Source: Biosensors (Basel). 2025 Jul 4;15(7):432. doi: 10.3390/bios15070432 (PMC12293830; doi:10.3390/bios15070432)

Table S1. Visual detection rates at serial CDV concentrations. Each concentration of CDV was tested in 5 replicates, except for $10^{2.39}$ $TCID_{50}$/0.1 mL, which was tested in 20 replicates. A visual positive was defined as the presence of a clearly visible test line as judged independently by three trained observers. The visual positive rate was calculated as the percentage of positive readings among total replicates. Mean score refers to the average visual intensity score assigned by observers on a scale from 0 (no line) to 2 (strong line). The visual limit of detection (LOD) was defined as the lowest concentration at which ≥95% of replicates showed visible bands , with an average score ⩾ 1.0 across replicates ($10^{2.39}$ $TCID_{50}$/0.1 mL in this case).

Virus concentration ($TCID_{50}$/0.1 mL)	No. of replicates	No. of visual positives	Visual Positive rate (%)	Mean Score
4.2	5	5	100%	2
3.6	5	5	100%	2
3.3	5	5	100%	2
2.99	5	5	100%	2
2.69	5	5	100%	1.87
2.39	20	19	95%	1.63
2.09	5	3	60%	0.73
1.79	5	1	20%	0.13
0	5	0	0%	0

Figure S1. Determination of the visual limit of detection (LOD) for CDV using serial dilutions. Visual detection rates and average visual scores for CDV at serial 2-fold dilutions. A visual positive was defined as a clearly visible test line evaluated independently by three observers. Each dilution was tested in five replicates, except for the critical LOD concentration ($10^{2.39}$ $TCID_{50}$/0.1 mL), which was tested in 20 replicates to improve statistical confidence. The blue dashed line represents the 95% visual positivity threshold. The red dashed line indicates the visual score threshold of 1.0, above which the test line was generally distinguishable by eye. The visual LOD was defined as the lowest concentration at which ⩾95% of replicates yielded a visually positive result.

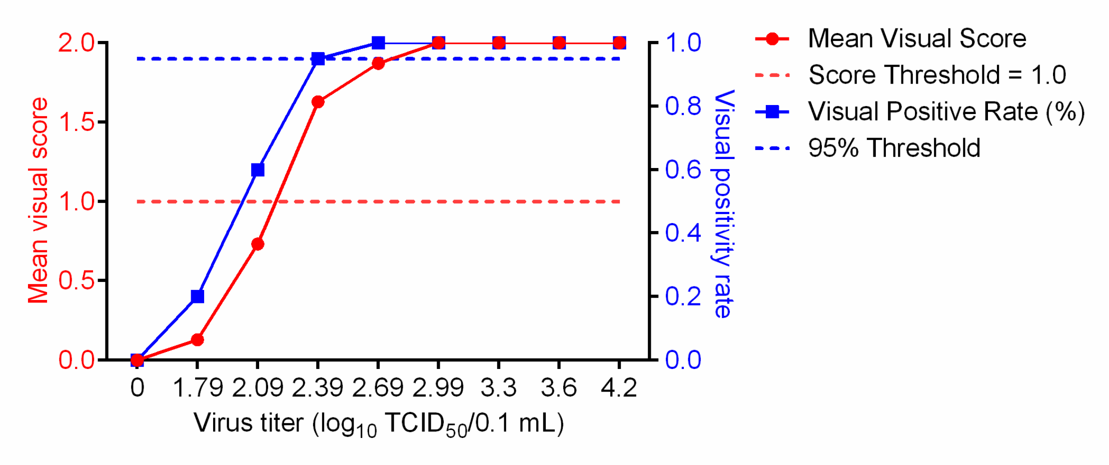

Supplement: Supplementary file 1 [file biosensors-15-00432-s001.zip › biosensors-3663535-supplementary.pdf]
